# An unusual intrathecal baclofen pump failure thirteen months after implantation

**DOI:** 10.1016/j.inpm.2023.100284

**Published:** 2023-10-14

**Authors:** Kevin E. Vorenkamp, Savan H. Gandhi, Andrew S. Manolides, Daniel T. Warren

**Affiliations:** a Virginia Mason Medical Center, 1100 9th Ave, Seattle, WA, 98101, USA; b ECU Health Medical Center, 2100 Statonsburg Rd, Greenville, NC, 27834, USA

**Keywords:** Intrathecal pump, Intrathecal drug delivery, Baclofen, Baclofen withdrawal, Intrathecal pump failure, Multiple sclerosis

## Abstract

**Objective:**

To report a case of intrathecal pump failure following months of diminishing benefit from intrathecal baclofen, and to heighten awareness that intrathecal pump malfunction can occur without precipitating events.

**Case report:**

A 40 year-old woman with multiple sclerosis and spastic paraplegia developed worsening spasticity after ten months of therapeutic stability with intrathecal baclofen. After other causes were pursued, this was discovered to be due to pump malfunction only thirteen months after implantation. After pump interrogation and discussion with the manufacturer the pump was replaced urgently and the patient regained therapeutic benefit and had no further complications**.**

**Conclusions:**

We present herein what we believe to be the first report of a verified pump malfunction resulting in disruption of intrathecal baclofen delivery within thirteen months of implantation. Due to the possible severity of acute baclofen withdrawal, the pump was replaced urgently after diagnosis. Because of the patient's and her healthcare providers' vigilance, she experienced no adverse events. Healthcare providers are encouraged to acknowledge the possibility of intrathecal pump malfunction in similar scenarios, ensuring patient safety while systematically examining the underlying problem.

## 1 Introduction

Intrathecal baclofen (ITB) infusion has demonstrated effectiveness for patients with severe spasticity refractory to conservative pharmacotherapy [[1], [2], [3], [4], [5], [6], [7]]. However, acute baclofen withdrawal can result in life-threatening sequelae that include high fever, altered mental status, exaggerated rebound spasticity, and muscle rigidity that in rare cases has advanced to rhabdomyolysis, multiple organ-system failure, and death [8,9]. Most commonly, baclofen withdrawal is a consequence of patient-related factors (missed refill appointment, pump with expired battery), practitioner errors (medication error, programming error, unintentional pump pocket refill), or disruption of the catheter and connections of the intrathecal drug delivery system (IDDS). Although inherent pump malfunction remains a possibility, it is generally felt to be an unlikely scenario that is not well reported within the medical literature. In 2014, there was a clinical study verifying the accuracy of intrathecal pump delivery rates [10]. However, in the same year there were two published cases of intrathecal (IT) pump failure resulting in an overdose of fentanyl [11]. We present herein what we believe to be the first report of a verified pump malfunction resulting in acute disruption of ITB delivery after a period of chronic under-delivery of baclofen without initial device alarm, and the case demands review for this reason. Internal review board approval was not required, but the patient did provide consent for publication of this case report.

## 2 Case report

A 40-year-old woman weighing 50 kg, with an 18-year history of multiple sclerosis and spastic paraplegia, underwent successful implantation of an ITB pump (Medtronic Synchromed® II, Minneapolis, USA). After implantation, the patient had good relief from her lower extremity spasticity for the initial twelve months of ITB therapy. She subsequently developed progressive worsening of spasticity which was unresponsive to escalating baclofen doses (from 316 μg (mcg) per day to 462 mcg per day over three dose modifications). Because of her worsening spasticity and concerns about the integrity of the pump-catheter system, a catheter dye study was performed which demonstrated excellent myelogram appearance with no evidence of catheter fracture or dye extravasation. Two days later, the patient reported hearing a multi-toned alarm every 10 min, signaling no medication was being delivered. During this timeframe, she denied further increased spasticity, any fevers, or delirium.

In response to the alarm, the patient presented to the emergency department. On initial examination, the patient appeared at baseline with notable spasticity in her lower extremities while sitting in her wheelchair. Her IT pump alarm was audible every 10 min, and interrogation of the device revealed a motor stall without recovery 6 h earlier, with stalls of 7 min and 60 minutes that occurred two and eight days previously. Multiple attempts to re-program the pump were unsuccessful. We consequently contacted experts through Medtronic technical support, who advised that multiple spontaneous motor failures in the past two weeks were indicative of a non-functioning pump. Although the patient was clinically stable, her pump was programmed to deliver a 50 mcg bolus, and she was re-started on her pre-implantation dose of oral baclofen, 20 mg (mg) every 6 h. She was scheduled for intrathecal pump replacement the next morning.

**Fig. 1:**
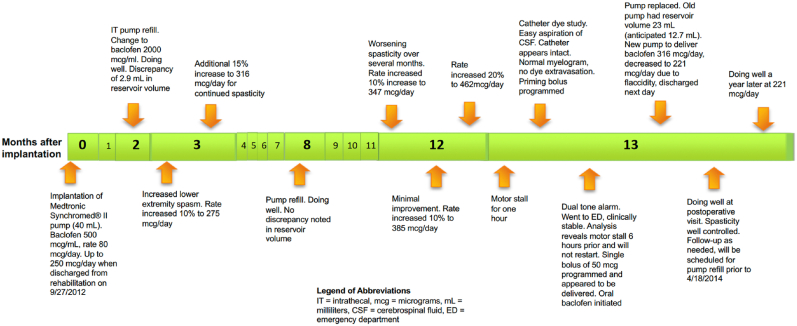
Timeline of Intrathecal Baclofen Delivery (in months after initial implantation).

At the time of surgery, no abnormalities were noted with the IT pump or catheter. After explantation of the non-functioning pump, 23 mL (mL) of baclofen solution was aspirated from the central reservoir rather than the expected 12.7 mL. Given this 10.3 mL discrepancy, her replacement pump was programmed to deliver baclofen at a rate of 316 mcg per day, which had been her stable dose prior to the escalating doses leading up to the pump replacement. After implantation, the patient noticed flaccidity along with mild sedation and therefore her rate was decreased 30 % to 221 mcg per day (See Fig. 1). Although we could not readily identify the dose delivered over the prior several weeks, when averaged over the time since her prior refill and the volume delivered, the average rate was calculated to be 213 mcg per day (17 mL of baclofen, 2000 mcg per mL, solution delivered over 160 days). At her prior refill 160 days earlier, there was no discrepancy between the volume expected and the volume aspirated from the central reservoir, and she had not yet developed increasing spasticity. She was discharged home in good condition the following day and continued to experience good control of her spasticity at a stable baclofen dose.

The patient's explanted pump was returned to the manufacturer, who provided this neuromodulation analysis summary:

“Analysis identified a motor feed through electrical short related to corrosion resulting in a motor stall. Analysis identified the 2-tone alarm was audible. Analysis identified the alarm volume was within specification. The Catheter Access Port (CAP) was found to be patent. Dispense accuracy testing could not be completed due to reset. The battery voltage tested within specification.”

## 3 Discussion

This clinical scenario represents a unique situation when an IDDS internally faulted, resulting in inadequate medication delivery and eventual failure. Fortunately, her pump eventually alarmed to this failure, and appropriate measures were taken to avoid serious complications. However, it seems that the pump had been under-delivering baclofen chronically long before any alarms were noted. The 23 mL aspirated from the reservoir was 10.3 mL more than the expected 12.7 mL, which is only accounted for by many days to weeks of under-delivery rather than just a few hours, which is how long the pump had been alarming. Unfortunately, the neuromodulation summary provided was “unable to test dispense accuracy.”

Abrupt cessation of ITB administration can cause baclofen withdrawal syndrome with the potential for serious consequences. Because of these concerns, her IT pump was replaced urgently the subsequent morning. Although she was not experiencing any withdrawal symptoms, she was provided an oral dose of baclofen to mitigate these concerns. Although oral baclofen cannot fully replace the anti-spasmodic effects of high-dose ITB, the patient was asymptomatic at the time. Additionally, the patient's pump was programmed to deliver an ITB bolus of 50 mcg. Although this bolus dose did appear to be delivered, given the status of the failing IDDS we discussed performing a single IT injection of baclofen if she experienced rebound spasticity or other withdrawal symptoms. There have also been reports of placing an external ITB delivery system when contraindications precluded placing a new IDDS [12].

ITB delivery is effective for severe spasticity, but withdrawal can result in the noted severe consequences. When patients present with worsening symptoms, physicians must focus on factors related to their underlying disease course and potential problems with the IDDS. At the time of the IT pump replacement, the large discrepancy in central reservoir volume provided a clear explanation of her worsening spasticity, as she had been receiving only a portion of her programmed dosing. This indicated that the ITB delivery was chronically inaccurate without any of this registering in the pump delivery log. This was further supported when the patient noted her spasticity was so greatly decreased upon administration via the new IDDS that she had overt flaccidity resulting in a needed decrease in her ITB dosage.

Although no similar cases of electrical short have been reported so soon after original implantation, Medtronic did issue a practice advisory in 2009 regarding possible premature battery failure in patients with an IDDS. They later sent a letter to physicians who implant pumps advising them to avoid using unapproved drugs in the Synchromed pump, stating that this could lead to intermittent or permanent motor stalls [13]. They had reported that use of unapproved medications increases the likelihood of pump failure from 0.9 % to 1.3 % at three years and from 2.4 % to 7.0 % at six years [14,15]. There has been another case with a similar mechanism of pump failure which occurred at 64 months post-implantation. In this case, an intermittent alarm was noted by the patient and the care team within 3 days of the alarm sounding. The patient was asymptomatic and did not require escalating baclofen requirements prior to pump replacement [16].

The case reported here is unusual in that it occurred so soon after implantation and with delivery of an approved medication. Another unique aspect of this case is the lack of alarm for which resulted in likely several weeks of chronic under-delivery of baclofen. Because of the patient's and her healthcare providers' vigilance, she experienced no adverse events. Healthcare providers are encouraged to acknowledge the possibility of IT pump malfunction in similar scenarios, ensuring patient safety while systematically examining the underlying problem.

## Declaration of competing interest

The authors declare that they have no known competing financial interests or personal relationships that could have appeared to influence the work reported in this paper.
